# Phytochemistry, Pharmacology, and Toxicology of *Datura* Species—A Review

**DOI:** 10.3390/antiox10081291

**Published:** 2021-08-15

**Authors:** Meenakshi Sharma, Inderpreet Dhaliwal, Kusum Rana, Anil Kumar Delta, Prashant Kaushik

**Affiliations:** 1 Department of Chemistry, Ranchi University, Ranchi 834001, India; meenakshiskkr@gmail.com (M.S.); akumardelta2013@gmail.com (A.K.D.); 2 Department of Plant Breeding and Genetics, Punjab Agricultural University, Ludhiana 141004, India; dhaliwalinderpreet@gmail.com; 3 Department of Biotechnology, Panjab University, Sector 25, Chandigarh 160014, India; kusumrana.86@yahoo.com; 4 Kikugawa Research Station, Yokohama Ueki, 2265 Kamo, Kikugawa City 439-0031, Japan

**Keywords:** *Datura stramonium*, alkaloids, atropine, cardiac glycosides, hyoscamine, Ayurveda

## Abstract

*Datura*, a genus of medicinal herb from the Solanaceae family, is credited with toxic as well as medicinal properties. The different plant parts of *Datura* sp., mainly *D. stramonium* L., commonly known as *Datura* or Jimson Weed, exhibit potent analgesic, antiviral, anti-diarrheal, and anti-inflammatory activities, owing to the wide range of bioactive constituents. With these pharmacological activities, *D. stramonium* is potentially used to treat numerous human diseases, including ulcers, inflammation, wounds, rheumatism, gout, bruises and swellings, sciatica, fever, toothache, asthma, and bronchitis. The primary phytochemicals investigation on plant extract of *Datura* showed alkaloids, carbohydrates, cardiac glycosides, tannins, flavonoids, amino acids, and phenolic compounds. It also contains toxic tropane alkaloids, including atropine, scopolamine, and hyoscamine. Although some studies on *D. stramonium* have reported potential pharmacological effects, information about the toxicity remains almost uncertain. Moreover, the frequent abuse of *D. stramonium* for recreational purposes has led to toxic syndromes. Therefore, it becomes necessary to be aware of the toxic aspects and the potential risks accompanying its use. The present review aims to summarize the phytochemical composition and pharmacological and toxicological aspects of the plant *Datura*.

## 1. Introduction

Medicinal plants present a wide range of bioactive substances known for their pharmacological activities. In fact, the majority of conventional medicines rely on plant products. One such plant species is *Datura* spp., a flowering medicinal herb that pertains to the *Solanaceae* family [1], primarily used as an intoxicant and hallucinogen [2]. It is widely cultivated in Europe, Asia, America, South Africa, and other tropical and subtropical regions [3]. *Datura* can be well-grown in average soils, but it prefers nutrient-rich and moist soil or alkaline soil [4]. Although the plant acts as a narcotic, it has distinct effects on human health, rendering it incredibly beneficial as medicine [5,6]. This may be attributed to the fact that it possesses antimicrobial, antidiabetic, anti-asthmatic, anti-inflammatory, antioxidant, analgesic, insecticidal, cytotoxic, wound healing, and neurological activities [7,8]. The *Datura* plant is also known for its larvicidal effects against red flour beetle (*Tribolium castaneum*) and mosquito repellent activities [9,10]. In addition, *Datura* spp. has also been used against animal bites such as snake bites, which helps relieve pain. *D. stramonium*, the well-known species of this family, is utilized for mystic and religious purposes along with its use as herbal medicine [11]. Moreover, *D. stramonium* seed is generally smoked to get a hallucinogenic experience [3].

The consumption of any part of *Datura* plant may lead to the severe anticholinergic effect that may cause toxicity. In fact, the entire plant is toxic to some extent, but the seeds are found to be the most toxic; neither drying out nor boiling destroys the toxic properties [12,13]. Ayurvedic system of medicine has described *D. stramonium* as a valuable therapy for numerous human illnesses such as wounds, ulcers, rheumatism, fever, inflammation, asthma, and toothache [3,14]. A leaf extract taken orally can treat asthma along with sinus infection, and stripped bark can heal burns, swellings, and ulcers when applied externally to the affected area [9,15]. However, in the modern system of medicine, the therapeutic potentials of *D. stramonium* are dominated by its toxic effects. The intake of large doses of *D. stramonium* disturbs the central nervous system and produces symptoms like confusion, hallucinations and amnesia, and bizarre behavior [16]. In addition, the signs and symptoms of acute *D. stramonium* poisoning include dryness of the lips and the epidermis, pupil dilation, urinary retention, impaired vision, and fast heartbeat [15,17].

Several incidences of accidental or intentional *D. stramonium* poisoning have been reported from different parts of the world, when eaten directly or through decoction made from herbal prescriptions, owing to its mind-affecting properties [18]. Therefore, the therapeutic applications require extensive research and analysis of the plant from every aspect, especially its toxicity. It should be consumed only with prior knowledge of its adverse effects since the consequences can be extremely harmful. With these facts, it is necessary to be aware of the toxic aspects and the potential risks accompanying its use.

As a result, a systematic review of the literature was conducted using keywords: datura; datura phytoconstituents; atropine datura; using the Preferred Reporting Items for Systematic Meta-Analysis (PRISMA) approach [19]. The present review aims to summarize the phytochemical composition, pharmacological and toxicological aspects of the plant *Datura*.

## 2. Ethnobotanical Description of *Datura*

The genus *Datura* is generally represented as annual or perennial herbs with glandular or more often simple hair [20]. Leaves of the plant are petiolate, having a simple leaf blade, and sinuate or completely dentate. They are approximately 10–20 cm in length and 5–18 cm in breadth, coated with soft and short greyish hairs [2]. The solitary flowers are found in inflorescences situated in leaf axils or branch forks. The larger flowers are generally actinomorphic with stout pedicels. The bracts, peduncles, and bracteoles are generally absent [20].

Further, the funnel-shaped and elongated corolla has cuspidate lobes. In many instances, elongated anthers are observed that are longitudinally dehiscing. The unevenly dehiscent, four valved fruit is discovered as a dry capsule that is unarmed and delimited usually by remnants of the persistent calyx [20]. The plants also consist of numerous seeds compressed laterally with a curved embryo and 12 pairs of chromosomes usually. Several species of *Datura* originated at different places globally. *D. stramonium* is an annual plant with an herbaceous base that is branched and glabrous, extending up to an approximate level of 1 m [13]. The branches are leafy, firm, erect, with pale yellow or green color, generally branched in a forked fashion. The top surface is greyish-green and dark, typically soft, while the lower surface is paler and minutely wrinkled when dehydrated. The calyx is very long, tubular, and swollen. The seeds are dark, flat, and kidney-shaped. Fruits are comparable to walnuts and filled with thorns (hence called “thorn apple”) [13].

## 3. Biochemical Composition of *Datura*

**Figure 1:**
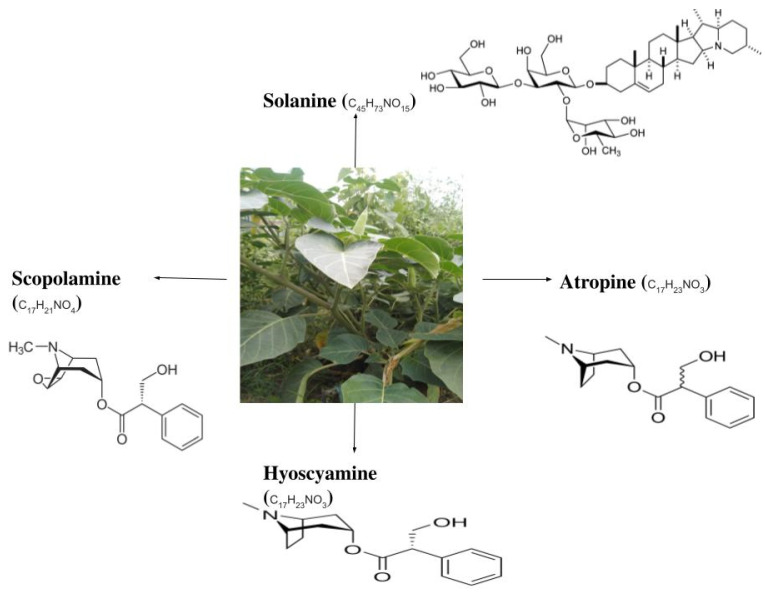
Identified important phytochemicals in *Datura*, as well as their chemical structures [30].

*Datura*, in general, constitutes significant amounts of carbohydrates, fats, protein, moisture, ash content, and crude fiber (Table 1). Besides, major phytochemicals found in *Datura* include alkaloids, phenolic compounds, tannins, flavonoids, and cardiac glycosides [21,22]. In addition, many amino acids such as alanine, phenylalanine, glutamate, and tyrosine have also been isolated from the seeds [8]. *Datura* species are particularly rich in tropane alkaloids. Hyoscine [(-)-Scopolamine] constitutes the major tropane alkaloid, along with hyoscyamine and atropine, having different concentration levels in different plant parts (Figure 1) [11,23]. The atropine content in the leaves in *Datura* *metel* was found to be 0.426%, whereas hyoscyamine levels were found to be 0.426% in the seeds and 0.43% in flower [8]. The alkaloid contents of scopolamine and atropine in the entire plant in *D.* *metel* increase gradually with the development of various growth stages and becomes most apparent when the plant reaches the end of its reproductive stage [24,25]. However, in the case of *D. stramonium*, the maximum amounts of alkaloids were found after ten weeks of seed germination, decreasing gradually with the beginning of the generative phase in plants [8,26]. Generally, the alkaloid concentration varies with the plant part and different growth stages in the plant. For example, leaves develop maximum alkaloid concentration in the vegetative phase, decreasing rapidly in the generative phase [27,28]. The stems and leaves of young plants contain hyoscyamine as a significant component. However, the concentrations of atropine and scopolamine differ in different plant parts in young and adult plants (Table 2) [9].

## 4. Pharmacological Activity of *Datura*

*Datura* is known to exhibit analgesic, antioxidant, anticancer, and antimicrobial properties. Especially, owing to the potent analgesic activities, *D. metel* acts as an effective painkiller. *D. stramonium* has antifungal activity against *Fusarium mangiferae* and *Fusarium oxysporum*, alkaloids found in *D. stramonium* are potential anticholinergic agents [31]. Atropine and scopolamine are muscarinic antagonists that may be utilized to cure Parkinson’s disease and parasympathetic stimulation of the eye, respiratory, urinary, heart, and gastrointestinal tract [32]. They prevent parasympathetic nerve impulses by selectively blocking the binding site of the neurotransmitter acetylcholine to the receptor of nerve cells [33]. In addition, *Datura* has long been utilized as a beneficial therapy for asthma symptoms. Atropine is the active anti-asthmatic agent that triggers paralysis of pulmonary branches of the lungs, removing the spasms responsible for the asthma attacks [34].

The technique of smoking *Datura* leaves through a pipe to alleviate allergies has its origins in the standard ayurvedic medicine in India. *D. stramonium* is utilized recreationally mainly for its anticholinergic consequences and can be produced by boiling the crushed seeds [13]. However, exposure of the fetus to *D. stramonium* causes a continuous release of acetylcholine, leading to desensitization of nicotinic receptors, resulting in permanent damage to the fetus [3].

### 4.1. Anti-Inflammatory and Analgesic Activities

The phytochemicals present in *Datura* species are well-known for their anti-inflammatory and analgesic properties due to their ability to suppress the production of chemical mediators responsible for the stimulation of nociceptors and induction of pain or inflammation [35,36]. The ethanolic extract of roots of *D.* *fastuosa* was found to exhibit anti-inflammatory activity when studied for paw edema induced by carrageenan in rats, with Indomethacin as a standard drug [37,38]. The progress of edema can be explained in different phases; release of histamine and serotonin in the initial phase, edema maintained by substances like kinin in the plateau phase, and prostaglandin release in the edema accelerating phase [37,38]. The root extracts showed considerable activity against inflammation at 200 mg/kg compared to Indomethacin (at 10 mg/kg). Moreover, the aqueous extracts of seeds and leaves possessed significant analgesic effects at 800 and 400 mg/kg dosage, respectively, when experimented on mice using a writhing test (induced by acetic acid) and hot plate reaction [8]. However, the analgesic activity induced by leaves could be potentially decreased by naloxone, while that of seed extract remained unaffected. The aqueous leaf extracts of *D.* *innoxia* have been evaluated for their anti-inflammatory activity to develop an active herbal pain-relieving drug [39]. The basic mechanism involved in anti-inflammatory and analgesic action is believed to be the cyclooxygenase (COX1 and COX2) inhibition, followed by suppression of prostaglandin-synthesis or probably the narcotic effects of *Datura* species [36,39].

### 4.2. Antioxidation Activities

The antioxidant activity of *Datura* extracts can also be attributed to the presence of phytochemical compounds, which act as potent free radical scavengers and help prevent cellular damage [40]. Analysis of *Datura* plant extracts for antioxidant characteristics revealed its ability to cure various health disorders, including cancers, since antioxidants are known to inhibit cell damage, the general pathway for cancers, aging, and several other diseases [41]. The antioxidant capacity of leaf extracts in different solvents, estimated by various in vitro methods, including hydroxyl radical scavenging activity, DPPH (2,2-diphenyl-1-picrylhydrazyl) scavenging activity, superoxide radical scavenging activity, β-carotene bleaching activity, and reducing power assay, revealed that chloroform extract of leaves possessed maximum concentration-dependent antioxidant activity [42,43]. The IC_50_ value of methanolic and hydroalcoholic seed extracts of *D.* *metel* recorded using the DPPH model showed that hydroalcoholic extract possesses slightly higher antioxidant activities (IC_50_ of 25.78 µg/mL) than methanolic seed extract (IC_50_ of 28.34 µg/mL) [7,42].

In the estimation of DPPH free radicals scavenging activities, a positive correlation was observed between the flavonoid and phenolic content of the *D.* *metel* extracts [8,42]. Maximum DPPH scavenging activity of methanolic seed extracts of *D. stramonium* was found to be 59.50% at a concentration of 60 μg/mL with IC_50_ value of 94.87 μg/mL; similarly, the maximum superoxide radical scavenging activity was 53.17% at a concentration of 60 μg/mL, while maximum scavenging activity of hydroxyl radical was 57.88% at concentration of 30 μg/mL with IC_50_ value of 39.59 μg/mL [41]. Moreover, the hydromethanolic root extract of *D. stramonium* exhibited a significant amount of correlation with DPPH free radical scavenging activity (Table 3).

### 4.3. Antimicrobial Potential of Datura

The antimicrobial activity against pathogenic microbes was evaluated using aqueous and ethanolic extracts of different plant parts of *D.* *stramonium*, and the results revealed that the ethanolic extracts showed better antimicrobial activity than the aqueous extracts [44,45]. Moreover, the leaf extracts were found to be more effective than stem and root. The branches and leaves of *Datura* *stramonium* extracted with different organic solvents such as benzene, chloroform, and ethanol exhibited significant antibacterial and antifungal activity when studied against *Enterobacter *sp.*, Micrococcus luteus*, *Pseudomonas aeruginosa*, *Escherichia coli*, *Staphylococcus aureus,* and *Klebsiella pneumonia* [8,46]. It was observed from the MBC (minimum bactericidal concentration) values that benzene extracts with the concentration of 3.12 mg/mL inhibited *P. aeruginosa* while the chloroform extracted with the same concentration was effective against *S. aureus*, *P. aeruginosa,* and *M. luteus*. Further, all the *D.* *stramonium* extracts were effective against various fungal strains such as *Aspergillus fumigatus*, *Aspergillus niger*, and *Saccharomyces cerevisiae* also, with maximum activity against *S. cerevisiae* and minimum antifungal activity against *A. niger* [8]. The methanolic and hydroalcoholic seed extracts of *D.* *fastuosa* also possessed considerable antimicrobial properties against bacterial (*Bacillus subtilis, Escherichia coli, Staphylococcus aureus*) and fungal (*Aspergillus niger* and *Candida albicans*) strains [8,20]. The methanolic extract acted against *E. coli* effectively with MBC of 25 µg/mL, while the hydroalcoholic extract was more effective against *B. subtilis* with both MBC and MIC (minimum inhibitory concentration) of 25 µg/mL. Methanolic plant extracts of *D. inoxia* also showed antifungal activity, and *Fusarium solani* was found more sensitive than other fungal species. The inhibition against different fungal species varied from 18.29 to 85.36%, where *F. solani* were affected more while *A. niger* showed more resistance [47]. *D. metel* seed oil had antibacterial activity against at least seven bacterial strains with the highest inhibition zone and lowest MIC against *Lactobacillus delbrueckii lactis* (19 mm) and *Pseudomonas aeruginosa* (18 mm), signifying susceptibility of bacterial strains to *D. metel* seed oil. The antibacterial activity exhibited a concentration-dependent response, and the inhibition zone increased with an increase in seed oil concentration [48].

### 4.4. Anti-Asthmatic and Bronchodilating Effects

Alkaloids found in *D.* *stramonium,* such as atropine and scopolamine, which possess significant anticholinergic and bronchodilating activities, block the muscarinic receptors (which are important for airways regulation), subsequently dilating bronchial smooth muscles [49,50]. Acetylcholine (the neurotransmitter synthesized and released by cholinergic neurons) leads to the contraction of smooth muscle after interaction with cholinergic muscarinic Acetylcholine (Ach) receptors [51]. The muscarinic receptors associated with the airway and lung tissues are M1, M2, and M3, of which M1 and M3, fully active in asthmatics, are responsible for bronchoconstriction while M2 which suppresses the release of acetylcholine, are less functional in asthmatics [52]. *Datura* administration inhibits M2 function, ultimately leading to the continued release of neurotransmitters. The anticholinergic activity of *D.* *stramonium* involves blocking the functions of muscarinic receptors on airway smooth muscles and submucosal gland cells [14]. The anti-asthmatic *D. stramonium* cigarette acts as a potential bronchodilator in asthmatic patients with the mild airway. A substantial decrease in the specific airway resistance (sRaw) was observed after inhaling smoke from the cigarette [8,53]. However, when exposed to the fetus during its use by the mother for asthma, *D. stramonium* releases acetylcholine. It desensitizes nicotinic receptors, resulting in slow or no response to repetitive agonist modulatory effects on the brain’s functioning, consequently damaging the fetus [3,13].

### 4.5. Anticancer Potentail of Datura

Chemical analysis of methanolic flower extracts of *D.* *fastuosa* resulted in the isolation of ten new withanolides, four of which possessed cytotoxic effects against various cancer cells, including BGC-823 (gastric), K562 (leukemia), and A549 (lung) [4,31]. The withanolides extracted from *D.* *fastuosa* when investigated for their cytotoxicity against human colorectal adenocarcinoma cells (DLD-1) and human lung carcinoma cells (A549)**,** 12α-hydroxy*Datura*metelin was found to exhibit cytotoxicity against DLD-1 and A549 cell lines, with corresponding IC_50_ values 2.0 and 7.0 µM [8,20].

Moreover, the root and stem extracts of *Datura* showed cytotoxic effects against human liver cancer cells (HepG-2) with IC_50_ values 613.88 and 341.12 µg/mL, respectively, while the leaf and root extracts are effective against cervical carcinoma cells (HeLa) with IC_50_ values of 267.76 and 348.35 µg/mL, respectively [8,20]. The MTT [3-(4,5-dimethylthiazol-2-yl)-2,5-diphenyltetrazolium bromide] assay method was used to carry out in vitro cytotoxicity assay in the Vero cell line; the IC_50_ value of methanolic fruit extracts against the Vero cell line was determined to be 3 mg/mL [54].

In addition, the seeds of *D. stramonium* are effective in treating gliomas, owing to their inhibitory effect on the proliferation of human glial tumor cells [55,56]. Moreover, malignancy of gliomas correlates inversely with the concentration of glial fibrillary acidic protein (GFAP). Interestingly, *D.* *stramonium* seed agglutinin were found to enhance the expression of GFAP, which provides them anticancer potential against gliomas. Experiments on cytotoxicity of the methanolic seed extracts of *D. Stramonium* on human-breast adenocarcinoma (MCF-7) cells revealed an increase in cytotoxicity with the increasing concentration of the extract with IC_50_ value of 113.05 μg/mL [41]. The ethanolic extracts of *D. innoxia* exerted cytotoxic effects on different cancer cells, especially against human colon cancer cells (LoVo), inducing cell-cycle arrest at the sub G1 phase and apoptosis [57].

### 4.6. Activity against Harmful Insects

Most of the plant extracts from *Datura* have insecticidal activity and help control the pest infestation. The methanolic seed extracts of *D.* *metel* are active against *Helicoverpa armigera* (Hubner), adversely affecting various development stages, including larval survival, pupal period, pupation percentage, and adult emergence [4,8]. In addition, the ethanolic leaf extracts of *D. stramonium* exhibited larvicidal and mosquito repellent properties against *Aedes aegypti* (86.25 µg/mL), *Anopheles stephensi* (16.07 mg/L), and *Culex quinquefasciatus* (6.25 mg/L), which is evident from the half lethal dose (LD_50_) values for larvicidal activity given in brackets, and complete protection time for mosquito repellency [3,58]. Further, the insecticidal activity of seed extracts of *D. stramonium* against *Sitophilus oryzae* in stored wheat grains was evaluated in different solvents [59]. Observations showed that acetone extracts exhibit the most potent activities (LD_50_ of 1.19 mL/kg), followed by chloroform extracts (LD_50_ of 7.38 mL/kg) and ethanol extract (LD_50_ of 8.59 mL/kg). Moreover, it was observed that longer intervals of time were required to obtain satisfactory levels of mortality. The mortality rate increased with increasing concentration from 4 mL/kg to 16 mL/kg and exposure time of 24 hrs and 96 hrs [59].

Furthermore, the extract from the different parts *D. stramonium* has shown the larvicidal and oviposition preventive potential against *Plutella xylostella* [60]. The highest toxicity against *P. xylostella* larvae was exhibited by extracts obtained from flowers (LC_50_ 82.3 µg/mL) followed by seeds (LC_50_ of 146.8 µg/mL), roots (LC_50_ of 165.3 µg/mL), leaves (LC_50_ of 526.6 µg/mL), and stem (LC_50_ of 841.4 µg/mL). Similarly, oviposition deterrent activity was highest with flower extracts (63%), while root (46%) and seed (44%) extract also produced significant results, which renders the extracts of flower, seed, and root highly effective against *P. xylostella* larvicidal as well as oviposition activity [60]. The investigation of toxic effects of *D. innoxia* acetone extracts against *Tribolium castaneum*, *Trogoderma granarium,* and *Sitophilus granarius* and the subsequent comparison of mortality rates showed that *D. innoxia* extracts exhibit remarkable lethal effects against these three insect species of stored grains and result in maximum mortality of 15.12%, 13.52%, and 14.07% in *T. castaneum*, *T. granarium*, and *S. granarius*, respectively [61]. Further a summary of activity of Datura of various plant parts of Datura species against harmful insects is presented in Table 4.

### 4.7. Cellular Protective and Wound-Healing Effects

The pre-treatment of rats with the seed extracts of *Datura* enhanced the survival rate after poisoning with organophosphate (dichlorvos), probably due to the presence of a significant amount of atropine in seeds and other anticholinergic compounds [17,62]. A dose of 7.5 mg/kg *Datura* seed extract (as a single intraperitoneal injection) prior to 25 mg/kg dichlorvos subcutaneous injection in male rats resulted in a significantly longer duration of survival with a median survival time of more than 24 h for the *Datura* treated animals [8,63].

Investigations on Wistar albino rats using the excision wound model demonstrated a positive correlation between the concentration of *D. fastuosa* ethanol extracts and wound-healing activities [8,64], examined based on several parameters, including the percentage of wound closure, the meantime for epithelization, hydroxyproline, DNA, and protein level. It was observed that *D.* *fastuosa* extracts (10% *w*/*w*) in the form of ointment possessed significant wound-healing capacity as compared to the standard Nitrofurazone ointment (0.2% *w*/*w*) [7,64].

## 5. Toxicology of *Datura*

In addition to a number of beneficial health outcomes, the presence of anticholinergic alkaloids such as tropane renders the *Datura* species toxic to the nervous system [6], and the symptoms of toxicity include fever, dry skin, dry mouth, headache, hallucinations, convulsions, rapid and weak pulse, acute confusion, delirium, tachycardia, coma, and death [4,8]. However, *D.* *fastuosa* was rendered safe up to a 2000 mg/kg body-weight dosage since it produced no sign of toxicity or mortality. Histological studies demonstrated the reduced weight of the organs, necrotic modifications in the liver accompanied by elevation of serum alkaline phosphatase, serum glutamic-oxaloacetic transaminase, and glutamyl pyruvic transaminase [65,66]. Moreover, due to its toxicity, *D.* *stramonium* should not be used in case of glaucoma, pyloric stenosis, paralytic ileus, tachycardia arrhythmias, enlarged prostate, and acute pulmonary edema [8]. The seed extracts at a concentration greater than 0.5% induced adverse physiological modifications. In fact, all the parts of *Datura* have severe anticholinergic effects due to suppression of central and peripheral cholinergic neurotransmission, ultimately leading to death in humans. Intoxication with *Datura* extracts leads to adverse impact on the central nervous system, disorientation, memory loss, inability to process information, impaired vision due to mydriasis, myoclonic jerking, hyperpyrexia, and respiratory and cardiovascular problems [3,67]. However, the aqueous extracts of leaves and seeds of *D.* *metel* demonstrated certain neurological effects in rats at doses of 400 and 800 mg/kg, which include enhanced motor activity in the brain, aggravated catalepsy, antagonized ptosis, and reduction in the duration of barbiturate induced sleep, in addition to its antidepressant characteristics at low doses [68]. The study also suggested that the seed extracts were comparatively safer to induce sleep with better anesthetic indices.

## 6. Conclusions

Plants and herbs have always been recognized as highly efficient and reliable medicines in modern and traditional medical systems. *D. stramonium* is among such medicinal herbs. A flowering plant, mostly found in the wild, *Datura* is a rich source of tropane alkaloids known for their anticholinergic activities, including atropine and scopolamine. They are extensively studied for their pharmacological properties, including decongestion of the respiratory system, stimulation of the central nervous system, treatment of skin infections, toothache, and other dental problems, and relief from pain. Although all the plant parts are toxic, the ripe seeds are found to contain the highest concentration of alkaloids. Thus, they can be effectively used to treat the symptoms observed due to organophosphate-induced toxicity and certain central anticholinergic effects. The leaf extract is taken orally to treat sinus infections, and the bark extract is applied externally to treat burns, ulcers, swellings, and other skin infections.

Owing to the medicinal properties of *Datura*, it has long been used to treat arthritis, aches, abscesses, headaches, rattlesnake bites, swellings, sprains, and tumors. Moreover, *Datura* has found its use in ayurvedic medicine for the treatment of wounds, inflammation, bruises and swellings, sciatica, ulcers, rheumatism, asthma, bronchitis, and body ache. It is also used as an ointment for reducing the pain of rheumatism and sciatica. The juice extracted from the leaves in the milk is highly effective in expelling intestinal worms such as cestodes.

The dried plant parts such as flowers, leaves, and roots were generally used as anti-asthmatic, antispasmodic, antitussive, bronchodilator, hallucinogenic, and narcotic agents. In addition, the plant is considered significantly important due to its antiphlogistic, antiseptic, anodyne, and sedative characteristics. Different parts of *D. stramonium* have been extensively used as a traditional remedy for various health disorders.

In addition to its use for numerous human ailments, *D. stramonium* is known for its toxicity also. Its toxic effects generally conceal its medicinal effects. Intoxication due to higher doses of *D. stramonium* causes severe damage to the central nervous system leading to an uncontrolled mental state. The other adverse impacts include hallucinations, convulsions, dryness in skin, irregular heart and pulse rates, memory loss, blurred vision, and coma, leading to death. Therefore, the pharmacological properties of *Datura* should be utilized with thorough knowledge of the possible toxicological outcomes to avoid its side effects.

## Figures and Tables

**Table 1:** Percentage composition of chemical compounds in the seeds of *D. metel, D. stramonium*, and *D. innoxia.*

Chemical Constituents	*D. metel* (%) [8]	*D. stramonium* (%) [28]	*D. innoxia* (%) [29]
Fat/lipid	14.72	16.60	15.52
Carbohydrate	51.22	26.20	-
Protein	20.73	16.20	13.90
Moisture	4.63	8.50	10.00
Ash content	5.14	8.70	8.26
Crude fibre	17.35	23.70	6.55

**Table 2:** Concentration (µg/mg) of atropine and scopolamine in different plant parts of *D. stramonium.*

Plant Parts	Alkaloid	Young Plant	Adult Plant
Stems	Atropine	0.915 ± 0.015	0.001 ± 0.001
	Scopolamine	0.129 ± 0.014	
Seeds	Atropine	0.670 ± 0.003	0.387 ± 0.015
	Scopolamine	0.012 ± 0.001	0.089 ± 0.010
Flowers	Atropine	0.299 ± 0.021	0.270 ± 0.026
	Scopolamine	0.106 ± 0.031	0.066 ± 0.004
Roots	Atropine	0.121 ± 0.015	-
	Scopolamine	0.014 ± 0.004	-
Medium leaves	Atropine	0.831 ± 0.014	0.150 ± 0.002
	Scopolamine	0.041 ± 0.005	0.022 ± 0.005

Source: [9].

**Table 3:** Concentration of hydromethanolic root extract of *D. stramonium* and its DPPH scavenging activity (%) [40].

Root Extract Concentration (μg/mL)	DPPH Scavenging Activity (%)
12.5	16.31
25	30.65
50	44.31
100	67.79
200	81.23
400	90.32

**Table 4:** Activity of different plant parts of Datura species against harmful insects.

Insect	Extract of Plant Part	Species	References
*Helicoverpa armigera*	Methanolic seed extracts	*D. metel*	[4,8]
*Aedes aegypti*	Ethanolic leaf extracts	*D. stramonium*	[58]
*Anopheles stephensi*	ethanolic leaf extracts	*D. stramonium*	[58]
*Culex quinquefasciatus*	ethanolic leaf extracts	*D. stramonium*	[58]
*Sitophilus oryzae*	Methanolic seed extracts	*D. stramonium*	[59]
*Plutella xylostella*	extract from the different plant parts	*D. stramonium*	[60]
*Tribolium castaneum*	Acetone extracts	*D. innoxia*	[61]
*Trogoderma granarium*	Acetone extracts	*D. innoxia*	[61]
*Sitophilus granarius*	Acetone extracts	*D. innoxia*	[61]
