# Correction: Sharma et al. Phytochemistry, Pharmacology, and Toxicology of *Datura* Species—A Review. *Antioxidants* 2021, *10*, 1291

**DOI:** 10.3390/antiox14020226

**Published:** 2025-02-17

**Authors:** Meenakshi Sharma, Inderpreet Dhaliwal, Kusum Rana, Anil Kumar Delta, Prashant Kaushik

**Affiliations:** 1 Department of Chemistry, Ranchi University, Ranchi 834001, India; meenakshiskkr@gmail.com (M.S.); akumardelta2013@gmail.com (A.K.D.); 2 Department of Plant Breeding and Genetics, Punjab Agricultural University, Ludhiana 141004, India; dhaliwalinderpreet@gmail.com; 3 Department of Biotechnology, Panjab University, Sector 25, Chandigarh 160014, India; kusumrana.86@yahoo.com; 4 Kikugawa Research Station, Yokohama Ueki, 2265 Kamo, Kikugawa City 439-0031, Japan

In the original publication [1], there was an error regarding the affiliation for author Prashant Kaushik. Specifically, affiliation number 5 (Instituto de Conservación y Mejora de la Agrodiversidad Valenciana, Universitat Politècnica de València, 46022 Valencia, Spain) has been removed from the affiliation list, as per the institution’s request. The institutional email for the author associated with the institution has also been changed with another email.

The authors state that the scientific conclusions are unaffected. This correction was approved by the Academic Editor. The original publication has also been updated.
